# Diagnostic accuracy of the Bedside Lung Ultrasound in Emergency protocol for the diagnosis of acute respiratory failure in spontaneously breathing patients*,**

**DOI:** 10.1590/S1806-37132015000100008

**Published:** 2015

**Authors:** Felippe Leopoldo Dexheimer, Juliana Mara Stormovski de Andrade, Ana Carolina Tabajara Raupp, Raquel da Silva Townsend, Fabiana Gabe Beltrami, Hélène Brisson, Qin Lu, Paulo de Tarso Roth Dalcin

**Affiliations:** Federal University of Rio Grande do Sul, Porto Alegre, Brazil. Graduate Program in Respiratory Sciences, Federal University of Rio Grande do Sul, Porto Alegre, Brazil; Ernesto Dornelles Hospital, Porto Alegre, Brazil. Intensive Care Unit, Ernesto Dornelles Hospital, Porto Alegre, Brazil; Ernesto Dornelles Hospital, Porto Alegre, Brazil. Intensive Care Unit, Ernesto Dornelles Hospital, Porto Alegre, Brazil; Ernesto Dornelles Hospital, Porto Alegre, Brazil. Intensive Care Unit, Ernesto Dornelles Hospital, Porto Alegre, Brazil; Federal University of Health Sciences of Porto Alegre, Porto Alegre, Brazil. Federal University of Health Sciences of Porto Alegre, Porto Alegre, Brazil; Université Pierre et Marie Curie, Paris, France, Hospital Practitioner. Multidisciplinary Intensive Care Unit (Prof. J.J. Rouby), Department of Anesthesiology and Critical Care, Pitié-Salpêtrière Hospital, Assistance Publique-Hôpitaux de Paris - AP-HP, Public Assistance-Paris Hospitals - Université Pierre et Marie Curie - UPMC, Pierre and Marie Curie University - Paris 6, Paris, France; Université Pierre et Marie Curie, Paris, France, Hospital Practitioner. Multidisciplinary Intensive Care Unit (Prof. J.J. Rouby), Department of Anesthesiology and Critical Care, Pitié-Salpêtrière Hospital, Assistance Publique-Hôpitaux de Paris - AP-HP, Public Assistance-Paris Hospitals - Université Pierre et Marie Curie - UPMC, Pierre and Marie Curie University - Paris 6, Paris, France; Federal University of Rio Grande do Sul, Department of Internal Medicine, Porto Alegre, Brazil, Associate Professor. Department of Internal Medicine, Federal University of Rio Grande do Sul, Porto Alegre, Brazil

**Keywords:** Ultrasonography, interventional, Respiratory insufficiency, Intensive care units

## Abstract

**Objective:**

Bedside lung ultrasound (LUS) is a noninvasive, readily available imaging modality that can complement clinical evaluation. The Bedside Lung Ultrasound in Emergency (BLUE) protocol has demonstrated a high diagnostic accuracy in patients with acute respiratory failure (ARF). Recently, bedside LUS has been added to the medical training program of our ICU. The aim of this study was to investigate the accuracy of LUS based on the BLUE protocol, when performed by physicians who are not ultrasound experts, to guide the diagnosis of ARF.

**Methods:**

Over a one-year period, all spontaneously breathing adult patients consecutively admitted to the ICU for ARF were prospectively included. After training, 4 non-ultrasound experts performed LUS within 20 minutes of patient admission. They were blinded to patient medical history. LUS diagnosis was compared with the final clinical diagnosis made by the ICU team before patients were discharged from the ICU (gold standard).

**Results:**

Thirty-seven patients were included in the analysis (mean age, 73.2 ± 14.7 years; APACHE II, 19.2 ± 7.3). LUS diagnosis had a good agreement with the final diagnosis in 84% of patients (overall kappa, 0.81). The most common etiologies for ARF were pneumonia (n = 17) and hemodynamic lung edema (n = 15). The sensitivity and specificity of LUS as measured against the final diagnosis were, respectively, 88% and 90% for pneumonia and 86% and 87% for hemodynamic lung edema.

**Conclusions:**

LUS based on the BLUE protocol was reproducible by physicians who are not ultrasound experts and accurate for the diagnosis of pneumonia and hemodynamic lung edema.

## Abstract (pt)

**Objetivo:**

O ultrassom pulmonar (USP) à beira do leito é uma técnica de imagem não invasiva e prontamente disponível que pode complementar a avaliação clínica. O protocolo *Bedside Lung Ultrasound in Emergency* (BLUE, ultrassom pulmonar à beira do leito em situações de emergência) demonstrou elevado rendimento diagnóstico em pacientes com insuficiência respiratória aguda (IRpA). Recentemente, um programa de treinamento em USP à beira do leito foi implementado na nossa UTI. O objetivo deste estudo foi avaliar a acurácia do USP baseado no protocolo BLUE, quando realizado por médicos com habilidades básicas em ultrassonografia, para orientar o diagnóstico de IRpA

**. Métodos:**

Ao longo de um ano, todos os pacientes adultos consecutivos respirando espontaneamente admitidos na UTI por IRpA foram prospectivamente inclusos. Após treinamento, 4 operadores com habilidades básicas em ultrassonografia realizaram o USP em até 20 minutos após a admissão na UTI, cegados para a história do paciente. Os diagnósticos do USP foram comparados aos diagnósticos da equipe assistente ao final da internação na UTI (padrão-ouro).

**Resultados:**

Foram inclusos na análise 37 pacientes (média etária: 73,2 ± 14,7 anos; APACHE II: 19,2 ± 7,3). O diagnóstico do USP demonstrou concordância com o diagnóstico final em 84% dos casos (kappa total: 0,81). As causas mais comuns de IRpA foram pneumonia (n = 17) e edema pulmonar cardiogênico (n = 15). A sensibilidade e a especificidade do USP comparado ao diagnóstico final foram de 88% e 90% para pneumonia e de 86% e 87% para edema pulmonar cardiogênico, respectivamente.

**Conclusões:**

O USP baseado no protocolo BLUE foi reproduzível por médicos com habilidades básicas em ultrassonografia e acurado para o diagnóstico de pneumonia e de edema pulmonar cardiogênico.

## Introduction

Acute respiratory failure (ARF) is a critical condition requiring dynamic evaluation and interventions. Bedside lung ultrasound (LUS) is a noninvasive, readily available imaging modality that can complement physical examination and clinical evaluation.^(^ 1 ^,^ 2 ^)^ The main advantage of bedside LUS is its immediate application to the diagnosis of thoracic disorders. Other advantages include delaying or even avoiding the need for patient transportation to the radiology suite or for radiation exposure and guiding life-saving therapies in extreme emergency.^(^ 1 ^,^ 3 ^-^ 5 ^)^ The use of LUS by emergency physicians, intensivists, and pulmonologists has been reported in many studies. ^(^ 1 ^,^ 4 ^-^ 10 ^)^

The appeal for using LUS in ARF patients is evident since LUS can detect lung aeration changes in many life-threatening conditions, such as acute lung edema, acute respiratory distress syndrome, pneumonia, and pneumothorax. ^(^ 4 ^-^ 6 ^,^ 10 ^-^ 14 ^)^ Recently, Lichtenstein and colleagues proposed a diagnostic algorithm-the Bedside Lung Ultrasound in Emergency (BLUE) protocol-to guide the diagnosis of severe dyspnea.^(^ 15 ^)^ The authors showed that the diagnostic accuracy of LUS, as measured against the final diagnosis made by the Intensive Care Unit (ICU) team, was 90.5%. Similarly, Silva et al. demonstrated that the diagnostic accuracy of the LUS approach in ARF patients was higher than was that of an initial routine evaluation based on clinical, radiological, and biological data (83% vs. 63%, p < 0.02).^(^ 16 ^)^

Since ultrasound is an operator-dependent imaging modality and bedside LUS is a recently developed tool, the reproducibility of findings obtained by physicians who are not ultrasound experts needs further validation. Indeed, the original BLUE protocol was performed by highly qualified ultrasound experts.^(^ 15 ^)^ Recently, ultrasound training has been added to the medical training program in our ICU. As we were concerned about the accuracy of bedside LUS performed by physicians who are not ultrasound experts, we therefore decided to investigate the diagnostic accuracy of the BLUE protocol for ARF.

## Methods

## Patients

We conducted a prospective study of all spontaneously breathing adult patients consecutively admitted to our 23-bed clinical-surgical ICU for ARF. This research was approved by the institutional ethics committee (Protocol no. 112/2011), which waived the requirement for informed consent.

The inclusion criteria were age ≥ 18 years and admission to the ICU for ARF, defined by one of the following: a respiratory rate ≥ 30 breaths/min; a PaO_2_ ≤ 60 mmHg; an oxygen saturation on room air ≤ 90%, as measured by pulse oximetry; or a carbon dioxide tension (PCO_2_) ≥ 45 mmHg with an arterial pH ≤ 7.35. The exclusion criteria were having required intubation before admission and/or having a multiple diagnosis or a rare (i.e., frequency < 2%) diagnosis, according to the original protocol.^(^ 15 ^)^

## Study design and LUS assessment

After attending 5 hours of theoretical training and performing 10 supervised LUS examinations, 4 non-ultrasound experts participated in the study. They were blinded to patient medical history and were not involved in diagnostic or therapeutic decisions. All patients were placed in a semirecumbent position and were evaluated with the same curvilinear probe with a range of 3-5 MHz (Toshiba Tosbee^(r)^; Toshiba, Tokyo, Japan). As a rule, LUS was performed within 20 minutes of admission, by one non-ultrasound expert. Each patient underwent a bedside chest X-ray at admission, which was interpreted by a radiologist unblinded to medical history. The initial clinical evaluation and diagnosis were performed by the physicians responsible for patient care. They were blinded to the LUS results but were aware of the chest X-ray results. The final diagnosis of the episode of ARF made by the ICU team before patients were discharged from the ICU was considered the gold standard. The main diagnoses, including pneumonia, acute hemodynamic lung edema, obstructive lung disease (i.e.*,* decompensated COPD or asthma), and pneumothorax, were evaluated. Patients with a multiple diagnosis or rare diseases were excluded from the analysis, as in the original BLUE protocol study.^(^ 15 ^)^

LUS images were recorded for each of six quadrants in each hemithorax (upper and lower parts of the anterior, lateral, and posterior chest wall, delimited by anterior and posterior axillary lines).^(^ 3 ^,^ 15 ^)^ Each quadrant was classified on the basis of worst findings into categories according to the predominant profile (A, B, or C) in each hemithorax, as previously described.^(^ 5 ^,^ 15 ^)^

**Figure 1 -:**
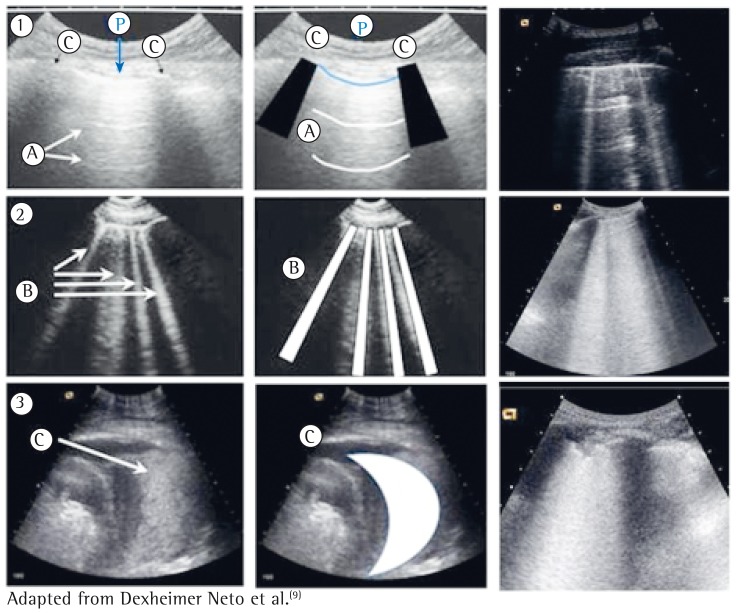
Lung ultrasound findings (left), their schematic representation (center), and illustrative examples (right). P: pleural line; c: ribs; A: A-lines; B: B-lines; and C: pulmonary consolidation.

The A, B, and C profiles were defined as follows (Figure 1):

- A profile (A-lines): white (hyperechoic) horizontal lines that are static and appear at regular intervals.
- B profile (B-lines): hyperechoic vertical artifacts that move in synchrony with the respiratory cycle.
- C profile: consolidation image appearing as a tissue structure containing white points consisting of lung parenchyma.

To identify normal lung aeration, lung sliding is a key ultrasound finding. It corresponds to the regular movement of the pleural line (described as a shimmering or bright white line) in regular cycles in synchrony with each respiratory movement. 

In accordance with the BLUE protocol (Table 1),^(^ 15 ^)^ a normal profile (bilateral lung sliding with A-lines) should be combined with screening for leg vein thrombosis. Deep venous thrombosis was sought using the same probe. A positive finding was the visualization of anatomic echoic intraluminal thrombosis or the absence of compression of femoral or popliteal veins. If there were signs of leg vein thrombosis, pulmonary embolism was the diagnosis; otherwise, the normal pattern was suggestive of respiratory dysfunction due to obstructive lung disease (i.e., decompensated COPD or asthma).^(^ 3 ^,^ 5 ^,^ 15 ^)^ The absence of lung sliding combined with the presence of A-lines was suggestive of pneumothorax. However, for this diagnosis, it was necessary to identify the lung point (the point where it is possible to identify both normal lung sliding and its absence). ^(^ 3 ^,^ 5 ^,^ 15 ^)^ Also, the identification of a normal anterior pattern associated with the presence of pleural effusion and posterior consolidation (A profile with posterior alveolar syndrome, pleural syndrome, or both) or an anterior or lateral consolidation (C profile) was suggestive of pneumonia.^(^ 3 ^,^ 15 ^,^ 16 ^)^

**Table 1 -:** Lung ultrasound profiles in acute respiratory failure.

Condition	Lung ultrasound finding
Pneumonia	AB profile, or consolidation, or A profile with posterior alveolar syndrome, pleural syndrome, or both
Acute hemodynamic lung edema	B profile
Obstructive lung disease (i.e., decompensated COPD or asthma)	A profile without DVT
Pneumothorax	A profile with a lung point and no lung sliding
Pulmonary embolism	A profile with DVT
DVT: deep vein thrombosis.	

DVT: deep vein thrombosis.

A B profile characterized by symmetric bilateral B-lines suggested hemodynamic lung edema. ^(^ 3 ^,^ 9 ^,^ 15 ^)^ However, B-line predominance without lung sliding could also be suggestive of pneumonia. 

The AB profile was characterized by asymmetric findings between the hemithoraces, suggestive of pulmonary infection as the etiology of ARF. 

## Statistical analysis

Categorical variables are expressed as numbers and percentages, and continuous data are expressed as mean ± standard deviation. The diagnostic performance of LUS as measured against each final diagnosis was assessed by calculation of sensitivity, specificity, and predictive values by using a standard formula. The completeness and accuracy of reporting was assessed with the Standards of Reporting of Diagnostic Accuracy checklist.^(^ 17 ^)^ In addition, the level of agreement among observers for the ultrasound findings and the final clinical diagnosis was evaluated with the kappa reliability test: kappa values < 0 indicated less than chance agreement; kappa values of 0.01-0.20 indicated slight agreement; kappa values of 0.21-0.40 indicated fair agreement; kappa values of 0.41-0.60 indicated moderate agreement; kappa values of 0.61-0.80 indicated substantial agreement; and kappa values of 0.81-0.99 indicated almost perfect agreement.^(^ 18 ^)^ McNemar's test was used to compare within-subject diagnostic accuracy between LUS and chest X-ray and between LUS and the initial clinical evaluation. Statistical analysis was performed with IBM-SPSS software, version 16 (IBM Inc., Armonk, NY, USA). All tests were two-tailed, and a p value < 0.05 was considered statistically significant.

## Results

Forty-two consecutive patients admitted to the ICU for ARF between October of 2011 and November of 2012 were enrolled in the study. As previously described, 5 patients with rare diagnoses were excluded from the final analysis (2 patients with pulmonary fibrosis, 1 patient with hypersensitivity pneumonitis, 1 with leptospirosis, and 1 with abdominal compartment syndrome). The baseline characteristics of the patients are shown in Table 2.

**Table 2 -:** Characteristics of the patients admitted to the ICU for acute respiratory failure (n = 37).a

Characteristic	Result
Age, years	73.2 ± 14.7
Male gender	16 (43)
BMI	25.7 ± 4.7
APACHE II score	19.2 ± 7.3
Glasgow Coma score	12.7 ± 3.1
pH	7.32 ± 0.13
PaO_2_/FiO_2_	173.15 ± 108.2
PCO_2_	50.9 ± 48
Previous diseases:	
Cancer	11 (30)
Heart disease	22 (59)
Heart Failure	7 (19)
Obstructive lung disease	8 (22)
Neurological disease	7 (19)
Chronic renal failure	4 (11)
Immediate trial of NIV	15 (41)
Success of NIV	6 (16)
Orotracheal intubation at admission (without a previous NIV trial)	19 (51)
Spontaneous breathing	3 (8)

BMI: body mass index; APACHE: Acute Physiological and Chronic Health Evaluation; PCO2: carbon dioxide tension; and NIV: noninvasive ventilation.

a Data expressed as mean ± SD or n (%).

Of the 37 medical patients, 70% were transferred from the medical ward. The mean hospital length of stay before ICU admission was 7.9 ± 7.7 days. Noninvasive or invasive positive-pressure ventilation was required in 92% of the patients (Table 2). The overall observed mortality was 42%.

According to the final diagnosis, the most common etiology of ARF was pneumonia (n = 17). Fifteen patients were admitted for hemodynamic lung edema, and 4 were admitted for obstructive lung disease. There was only one patient with pulmonary embolism (in this patient, LUS was normal as expected, but it was not possible to identify deep vein thrombosis) and none with pneumothorax. The sensibility, specificity, and predictive values are shown in Table 3. Pulmonary embolism and pneumothorax were not included because the number of patients with these conditions was insufficient to perform diagnostic performance analysis.

**Table 3 -:** Diagnostic performance of bedside lung ultrasound for each diagnosis.

Diagnosis	Sensitivity	Specificity	Positive predictive value	Negative predictive value
Pneumonia (n = 17)	88%	90%	88%	90%
Hemodynamic lung edema (n = 15)	85%	87%	80%	91%
Obstructive lung disease (n = 4)	67%	100%	100%	94%

The BLUE protocol diagnosis made at admission by physicians who are not ultrasound experts had a perfect agreement with the final diagnosis in 84% of the patients (overall kappa, 0.81). Agreements between the 2 methods were 0.78 and 0.74 for pneumonia and lung edema, respectively. 

The diagnostic accuracy of LUS alone was significantly higher than was that of chest X-ray alone (84% vs. 43%; p = 0.01). No significant difference was found between LUS and the standard initial clinical evaluation (84% vs. 65%; p = 0.12).

## Discussion

The main result of the present study is that bedside LUS performed by physicians who are not ultrasound experts allows the correct diagnosis of the most common causes of ARF (pneumonia and hemodynamic lung edema) with good sensitivity and specificity, as measured against the final diagnosis. The high overall diagnostic accuracy of LUS (84%) and the good agreement (kappa coefficient, 0.81) between LUS and the final diagnosis confirmed the high diagnostic yield of LUS. Indeed, the diagnostic accuracy of LUS was higher than was that of chest X-ray. 

The primary concern that led us to perform the present study was operator bias, since different operators could interpret ultrasonographic patterns of lung differently. Gaining competence in a skill over time is a well-recognized process, which has also been demonstrated for LUS.^(^ 19 ^)^ In most previous studies, a limited number of investigators who were experts in LUS performed the ultrasound examinations.^(^ 10 ^,^ 15 ^,^ 16 ^)^ Lichtenstein et al. reported a sensitivity and a specificity of 97% and 95%, respectively, for hemodynamic lung edema and of 94% and 89%, respectively, for pneumonia.^(^ 15 ^)^ In our study, the values obtained by physicians who are not ultrasound experts, although slightly lower (86% and 87%, respectively, for lung edema, and 88% and 90%, respectively, for pneumonia), are close to those reported by expert physicians. ^(^ 10 ^,^ 15 ^)^ This result indicates that the BLUE protocol is feasible and reproducible. 

Recently, Silva et al. compared the accuracy of cardiothoracic ultrasound with that of an initial clinical evaluation, as measured against the final diagnosis made by an expert panel, in 78 ARF patients.^(^ 16 ^)^ The authors found that the ultrasound approach was significantly more accurate than was the initial clinical approach (83% vs. 63%, respectively; p < 0.02).This finding indicates that the use of LUS data could have significantly improved the initial diagnosis.^(^ 16 ^)^ Similarly, it has been shown that therapeutic management can be changed directly as a result of information provided by LUS in up to 47% of mechanically ventilated patients.^(^ 20 ^)^ Interestingly, our results show similar accuracy rates, with accuracy being higher for LUS than for the initial clinical evaluation (84% vs. 65%).^(^ 16 ^)^ However, our study was underpowered to find a significant difference. 

In this study, LUS accuracy was significantly higher than was that of chest X-ray (84% vs. 43%; p = 0.009). Indeed, bedside LUS has been shown to have superior accuracy when evaluating patients with atelectasis, pneumothorax, pneumonia, or acute respiratory distress syndrome, compared with chest X-ray.^(^ 2 ^,^ 4 ^,^ 9 ^,^ 14 ^,^ 21 ^)^

In an attempt to increase concordance, all patients were evaluated in the same position and with the same probe. There is no recommendation for the duration of LUS training.^(^ 22 ^-^ 24 ^)^ In the present study, in order to homogenize the interpretation of LUS findings, we arbitrarily chose a total of 5 hours of theoretical training and 10 supervised ultrasound examinations. With this training method, our operators were able to individually achieve substantial diagnostic agreement (kappa coefficient, 0.81).

Bedside LUS is rapidly becoming integral to the evaluation of critically ill patients. However, it is still not widely used in Brazil. Costs are often regarded as major barriers.^(^ 22 ^)^ In a study conducted in Italy, the use of bedside LUS was associated with a 26% reduction in the total number of chest X-rays and a 47% reduction in the total number of CT scans.^(^ 25 ^)^

The main limitations of this study are its small sample size and the fact that it was conducted in a single center. Because our results are based mainly on the diagnoses of pneumonia and hemodynamic lung edema, further studies are needed to validate the BLUE protocol in the diagnosis of other causes of ARF. In addition, intra- and inter-operator variabilities were not assessed. Furthermore, as we followed the original BLUE protocol, our study did not incorporate the diagnosis of pleural effusion as an etiology of ARF, although LUS has great potential in the diagnosis of this pattern.^(^ 5 ^)^

In conclusion, this study, conducted in an ICU in Brazil, has demonstrated that the BLUE protocol is feasible and can easily be implemented in the ICU. After a brief training period, physicians are able to diagnose the main causes of ARF with accuracy.
